# A cross-sectional survey of policies guiding second stage labor in urban Japanese hospitals, clinics and midwifery birth centers

**DOI:** 10.1186/s12884-016-0814-2

**Published:** 2016-02-24

**Authors:** Kaori Baba, Yaeko Kataoka, Kaori Nakayama, Yukari Yaju, Shigeko Horiuchi, Hiromi Eto

**Affiliations:** St. Luke’s International University, Tokyo, Japan; Nagasaki University, Nagasaki, Japan

**Keywords:** Obstetric labor, Childbirth, Policy, Second labor stage, Midwifery, Guideline, Hospital, Clinic, Midwifery birth center, Cross-sectional survey

## Abstract

**Background:**

The Japan Academy of Midwifery developed and disseminated the ‘2012 Evidence-based Guidelines for Midwifery Care (Guidelines for Midwives)’ for low-risk births to achieve a more uniform standard of care during childbirth in Japan. The objective of this study was to cross-sectional survey policy implementation regarding care during the second stage of labor at Japanese hospitals, clinics, and midwifery birth centers, and to compare those policies with the recommendations in Guidelines for Midwives.

**Methods:**

This study was conducted in the four major urbanized areas (e.g. Tokyo) of the Kanto region of Japan. Respondents were chiefs of the institutions (obstetricians/midwives), nurse administrators (including midwives) of the obstetrical departments, or other nurse/midwives who were well versed in the routine care of the targeted institutions. The Guidelines implementation questionnaire comprised 12 items. Data was collected from October 2010 to July 2011.

**Results:**

The overall response was 255 of the 684 institutions (37 %). Of the total responses 46 % were hospitals, 26 % were clinics and 28 % were midwifery birth centers. Few institutions reported perineal massage education for ‘almost all cases’. Using ‘active birth’ were all midwifery birth centers, 56 % hospitals and 32 % clinics. Few institutions used water births. The majority of hospitals (73 %) and clinics (80 %) but a minority (39 %) of midwifery birth centers reported ‘not implemented’ about applying warm compress to the perineum. Few midwifery birth centers (10 %) and more hospitals (38 %) and clinics (50 %) had a policy for valsalva as routine care. Many hospitals (90 %) and clinics (88 %) and fewer midwifery birth centers (54 %) offered hands-on technique to provide perineal support during birth. A majority of institutions used antiseptic solution for perineal disinfection. Few institutions routinely used episiotomies for multiparas, however routine use for primiparas was slightly more in hospitals (21 %) and clinics (25 %). All respondents used fundal pressure as consistent with guidelines. Not many institutions implemented the hands and knees position for correcting fetal abnormal rotation.

**Conclusions:**

This survey has provided new information about the policies instituted in three types of institutions guiding second stage labor in four metropolitan areas of Japan. There existed considerable differences among institutions’ practice. There were also many gaps between reported policies and evidence-based Guidelines for Midwives, therefore new strategies are needed in Japan to realign institution’s care policies with evidenced based guidelines.

## Background

Assuring clinically effective and safe birth is of utmost importance worldwide. A high quality of care is needed for all expectant mothers to ensure a safe delivery. Even in countries with well-established health care infrastructures such as Japan, following evidence-based clinical guidelines could prevent large disparities and gaps in practice among the institutions providing maternity care [1].

Clinical practice should be guided by appropriate policies and toward that end several evidence-based guidelines had a major impact on Japan’s policies [2]. In 1996, WHO published ‘Care in Normal Birth: A Practical Guide’, which included evidence-based recommendations for routine care for women during low-risk labor and childbirth [3]. Later in 2007, The National Institute for Health and Clinical Excellence(NICE), operating in England, produced their Guidelines about perinatal care: ‘Intrapartum Care: care of healthy women and their babies during childbirth’ [4].

In Japan, three clinical Guidelines about intrapartum care were published: (1) ‘Evidence-based Guidelines for Comfortable Pregnancy and Childbirth [5]’, (2) ‘Guidelines for Maternity Home Services [6]’ and (3) ‘Clinical Guidelines for Obstetrical Practice in Japan 2011 edition [2]’; however, none adequately addressed the care of low-risk births. For example, ‘Clinical Guidelines for Obstetrical Practice’ published by the Japan Society of Obstetrical Practice [2] is mainly for high-risk women who may require medical intervention during pregnancy and delivery. Furthermore, ‘Evidence-based Guidelines for Comfortable Pregnancy and Childbirth’ [5] was mainly for low-risk women, and did not include necessary evidence-based recommendations for midwives concerning preventive care or the use of drugless interventions for low risk pregnancy and delivery. At the same time, there were not many reports documenting care policies of Japanese institutions and those reports were outdated, so that either care for low-risk births was not standardized, or there were many gaps between policies and international guidelines in Japan [7–12]. Accordingly, we needed to develop evidence-based guidelines for low-risk births for midwifery care, and to survey Japanese care policies.

Therefore the Japan Academy of Midwifery developed the ‘2012 Evidence-based Guidelines for Midwifery Care during Childbirth (Guidelines for Midwives)’ [13] for low-risk births in 2012. Although the target audience for the Guidelines for Midwives [13] was midwives, other medical personnel, concerned for the care of women, were also included. The contents of the Guidelines for Midwives [13] contain evidence-based care policies, which are necessary for clinical practice based on research derived from the clinical expertise of midwives. The data were drawn from primary and secondary documents identified from three databases: Cochrane Library through October 2012, and PubMed, Ichushi-Web through October 2008, plus three Guidelines: ‘Intrapartum Care: Care of Healthy Women and their Babies during Childbirth [4]’ from England; Japanese ‘Guidelines for Obstetrical Practice in Japan 2011 edition [2]’ and ‘Evidence-based Guidelines for Comfortable Pregnancy and Childbirth [5]’.

The Guidelines for Midwives are applicable to the three types of institutions for birthing in Japan: hospitals, obstetrician’s clinics and midwifery birth centers. In 2011 the combined total births from hospitals was 52 %, at obstetrician’s clinics 47 %, and midwifery birth centers and home births was 1 % [14]. The obstetrician’s clinics and hospitals in Japan differ based on the number of beds, with hospitals having more beds. Most midwives work at obstetricians’ clinics or hospitals. Approximately 5 % of midwives work at midwifery birth centers and are independent midwives. Practice at midwifery birth centers in Japan is restricted to low risk pregnancies. Midwives’ practice spans the management of antenatal care throughout pregnancy, birth and the postpartum period and is regulated by the Act for Public Health Nurses, Midwives, and Nurses [15].

No research has been required about comparing the policies among these institutions in Japan. Even so, recent research has documented the policies guiding the first and third stage of labor in the three types of institutions [16, 17]. There were many gaps between the policies and Guidelines for Midwives [16, 17]. Little is known about the types of operational policies which hospitals, clinics and midwifery birth centers have adopted to guide the second stage of labor. That data will be useful for working on a plan for improved second stage labor in Japan. The objectives of this study were to survey policy implementation regarding care during the second stage of labor in hospitals, clinics, and midwifery birth centers in Japan, and to compare the policies with the recommendations by Guidelines for Midwives.

## Methods

### Design, participants and settings

This study, a cross-sectional survey, was part of a larger study regarding the diffusion of the Guidelines for Midwives [16, 17]. It was conducted in the highly developed and urbanized Kanto region of Japan, which includes Tokyo, Kanagawa, Saitama and Chiba. Tokyo is the capital of Japan and has the largest population (13 million) followed by the contiguous regions of Kanagawa (9 million), Saitama (7 million) and Chiba (6 million). Invited to participate were all institutions, a total of 684 which had an obstetric ward dealing with delivery in these regions. They were identified by searching the internet, NTT yellow pages [18], the hospitals and clinic list [19] and the national midwifery birth centers map [20] for hospitals, clinics and midwifery birth centers. We mailed the consent form to managers in charge of hospitals, clinics, and midwifery birth centers. We made a reminder telephone call to all managers who had not replied to confirm if they were still willing to participate. Respondents were chiefs of the institutions (obstetricians/midwives), nurse administrators (including midwives) of the obstetrical department, or other nurse/midwives who were well versed in the routine care of the targeted institutions. The survey was conducted from October 2010 to July 2011.

### Measurements

#### Questionnaire development

All questions included in the questionnaire were derived from the Guidelines for Midwives [13] regarding routine obstetric care policies and practices for low-risk pregnancies. Guidelines for Midwives [13] was constructed from clinical questions about obstetric care from the first stage of labor to the early postpartum period, and the questions from the questionnaire relating to the clinical questions in Guidelines for Midwives [13] were included. Guidelines for Midwives [13] was structured based on the evidence and interpretation of each clinical question. Table 1 displays the evidence and interpretation of each clinical question of the Guidelines for Midwives [13]. Researchers Nakayama and Kataoka, both midwives, developed the questionnaire draft after which the remaining researchers used a process of discussion and consensus to make modifications. The co-researchers had been involved in the preparation of the Guidelines for Midwives [13], so they were well acquainted with its contents. Three expert clinical midwives established face validity through discussion and consensus.

**Table 1:** The Clinical Questions and Evidence of ‘2012 Evidenced-based Guidelines for Midwifery Care’ developed by the Academy of Midwifery

Clinical Questions	Evidences
CQ15. Perineal cleansing.	There is no evidence of using benzalkonium chloride or Chlorhexidine for vulva sterilization. One controlled study from the UK compared using cetrimide/chlorhexidine to using just tap water for perineal cleansing during labor. The study found no significance between the number of women who developed fever, used antibiotics, had perineal infection, and intention of perineal tear. Also, there was no significant difference in the outcomes of the babies. Therefore, this study’s evidence indicates that using cetrimide/chlorhexidine in no more effective than tap water for perineal cleansing.
CQ16. Position in the second stage of labor.	There are benefits and risks of each position, but there is no specific evidence that the supine position is more beneficial for women. There is high-level evidence that, when compared to spine position, upright position significantly reduced labor duration; the occurrence of vaginal instrumental birth; and it decreased episiotomy, pain and the incidence of fetal heart rate abnormalities. On the other hand, a high percentage of women using the up-right position had second-degree perineal tear and blood loss of over 500ml. There is no significant difference in third or fourth degree perineal tear for women in the upright position compared to women in the supine position. A woman should be informed about both the benefits and risks of each position and she should be able to choose the position. There is no specific evidence for position other than spine position is beneficial. Also there is no evidence for safeness. If there is a possibility of an abnormal labor then the spine position was recommended.
CQ17. Effectiveness of fundal pressure during the second stage of labour.	There is no evidence available on the effects of manual fundal pressure. Fundal pressure by an insufflatable belt during the second stage of labour does not appear to shorten the second stage of labor. Several reports suggest that fundal pressure is associated with maternal and neonatal complications such as uterine rupture, neonatal fractures and brain damage. Also anal sphincter damage has been reported. In the “Clinical Guidelines for Obstetrical Practice”, they recommended recommended complementary use of fundal pressure for vacuum extraction or forceps but with caution. Fundal pressure during the second stage of labor is not recommended for normal delivery.
CQ18. Effectiveness of perineal massage during the second stage of labor.	There is no evidence that perineal massage is effective in reducing the incidence of perineal tears or episiotomies. The only significant difference was that third-degree tears occurred less frequently in the group using perineal massage. Another RCT compared using perineal massage to applying warm compresses to the perineum, massaging the perineum with oil, and not touching the perineal until the baby’s head crowns during second stage of labor. This RCT found no significant difference in the incidence of perineal tear or episiotomy between the study groups. In addition, the women in the group who used perineal massage were the most willing to stop the intervention, yet there was still no significant difference in the incidence of third-degree perineal tear in the massage group compared to the other groups. Since there was no evidence that perineal massage prevents perineal tears, perineal massage should not be performed by health care professionals during the second stage of labor.
CQ19. The effectiveness of applying warm compresses to the perineum in order to prevent perineal tear during the second stage of labor.	There was no evidence that applying warm compresses to the perineum is effective for preventing perineal trauma. However, there was evidence that the group using the warm compresses experienced less perineal pain in post-delivery day one and two than the other group. NICE guideline indicates from a cohort study that perineal trauma occurs less frequently when a warm compress is applied to the perineum. In a RCT intended for nulliparas, the women in the warm compress group were less likely to have third-degree perineal tears compared to the women in the control group. However, NICE evaluated one US RCT and found that there was no significant difference in the incidence of perineal trauma between the groups applying warm compress to the perineum, perineal massage with oil, and the group not touching the baby’s head until it crowned during the second stage of labor. Since different studies show conflicting information there is no evidence that supports applying warm compresses to the perineum in order to prevent perineal tear. However, no outcomes of harm occurred and it was found to decrease pain during labor and post-delivery day one and two; warm compress to the perineum can be one option.
CQ20. Hand position during the birth of a baby.	There was no evidence of the effectiveness of two different methods of perineal management used to prevent perineal tears during delivery in the lateral position. In the NICE guideline, there was limited high-level evidence that women allocated to a ‘hands on’ perineal management group reported less pain at ten days after delivery compared to those women allocated to a ‘hand poised’ group. Also, in the ‘hand poised’ group, a smaller percentage of episiotomies were conducted in the ‘hand poised’ group compared to the ‘hands on’ group. There was no difference in the incidence of perineal trauma between the two groups and both methods of perineal management could be useful. There was no significant difference in the other RCT. However, the three studies used for the NICE guideline did not take delivery position into consideration during analyses and the RCT only considered the lateral position. Not to mention, the incidence of perineal injury differed by race. Therefore, there is no significant difference between two groups who were in lateral position. However, further study is needed to take other factors into consideration such as labor positions, race, and delivery environment. Since no study has been conducted in Japan, further study is needed.
CQ21. Routine vs. restricted use of episiotomy.	There is evidence that restrictive use of episiotomy is more beneficial to women when compared to those women in the routine episiotomy group. The results of one systematic review showed that 75 % of women had episiotomies in the routine group while 28 % of women in the restrictive group had episiotomies. Obviously there was lower incidence of episiepisiotomy in the restrictive group. There was a small percentage of women with severe perineal trauma who needed suturing or who experienced the complication of dysraphism in restrictive group. There was no significant difference in the outcomes of vaginoperineal trauma, dyspareunia, urinary incontinence, perineum pain, or asphyxia of newborn. Therefore, restrictive use of episiotomy is more beneficial to both women and babies. NICE guideline only recommends restrictive use of episiotomy when it is needed for an instrumental delivery or for fetal abnormality, but not for routine use. In conclusion, episiotomy is not needed for all women. Rather, restrictive use is recommended when it is medically necessary during low-risk delivery in Japan.
CQ22. Effectiveness of taking hands and knees position for correcting fetal abnormal rotation during progressing labor.	There is no obvious evidence that the hands and knees position fixes the abnormal rotation of the fetus or that it is effective in relieving the back pain that comes from abnormal rotation. There have been a few research studies on the effectiveness of the hands and knees position for abnormal rotation, but only one study was used for NICE guideline and a Cochrane systematic review. According to this study, there was no significance in the number of babies fixed as occipitoposterior to position occipitoanterior presentation. However, there is a tendency to correct the fetal abnormal rotation. In addition, this position was effective for relieving back pain and many women wanted to use the hands and knees position in her next delivery. No harm to mother or fetus has been reported due to being in the hands and knees position.

#### Question items about care during the second stage of labor

The survey comprised 12 items related to clinical questions (CQ15 – CQ 22) of the Guidelines for Midwives [13] about care during the second stage of labor. The items as worded do not imply that it is an expected practice but a practice that may or may not be in effect at the participants’ institution. The 12 items are: perineal massage education for pregnant women; ’active birth’ during the second stage of labor; ’active birth’ at delivery; water birth; applying warm compress to the perineum in order to prevent perineal tearing during the second stage of labor; Valsalva (closed glottis) during the second stage of labor; hands-on technique to support fetal expulsion; perineal disinfection; episiotomy for primiparas; episiotomy for multiparas; fundal pressure during the second stage of labor and hands and knees position for correcting fetal abnormal rotation during the second stage of labor. The definition of Valsalva maneuver is pushing as long as possible with closed glottis and is based on the definition in Evidence-based Guidelines for a comfortable pregnancy and childbirth [5].

For most items respondents selected one of three choices: (a) almost all cases, (b) depending on the case or (c) not implemented. On the other items they selected from two choices: (d) implemented or (e) not implemented. When the answer ‘almost all cases’ or ‘depending on the case’ or ‘implemented’ was selected for any or all of four items (perineal disinfection, episiotomy for primiparas, episiotomy for multiparas, or fundal pressure during the second stage in labor), respondents were to answer an additional question: in what cases/how were care policies implemented. Furthermore, for hands and knees position for correcting fetal abnormal rotation during the second stage of labor, respondents were asked to include the length of time women remained in the position.

Questions about the characteristics of each institution were included: the type of institution (hospital, clinic, midwifery birth center); the number of vaginal deliveries per year; the number of midwives and the type of obstetrics unit including only obstetrics or obstetrics and gynecology unit, or other such as mixed patients unit.

### Data analysis

Data analyses were conducted using IBM SPSS Statistics version 19.0. The statistical quantities were described and frequency distributions were calculated for the responses to each question. Data analyses included chi square test or Fishers’ exact test, and values of *P* < .05 were considered statistically significant. Non-responses were excluded from the analyses.

### Ethical considerations

The anonymous survey questionnaire was sent to all 684 institutions. An attached letter included these ethical considerations: the purpose of the survey, an assurance of the protection of privacy and a statement that participation was voluntary. Return of the questionnaire indicated their agreement to participate in the survey. The Ethics Committee of St. Luke’s International University, Tokyo, Japan approved this study (No. 10–1002).

## Results

### Characteristics of participating institutions

The response rate of the 684 institutions receiving questionnaires was 255 (37.3 %), of which 46.3 % (118/255) were hospitals, 25.9 % (66/255) were clinics and 27.8 % (71/255) were midwifery birth centers. However, by type of institution and the percent responding were 118 hospitals (50.2 %), 66 clinics (20.8 %) and 71 independent midwifery homes/clinics (54.2 %). The average number of vaginal deliveries per year was: 737.7 ± 528.1 at hospitals, 371 ± 288.7 at clinics, and 37.3 ± 44.7 at midwifery birth centers. The average number of midwives was 21.9 ± 17.9 at hospitals, 7.2 ± 5.1 at clinics, and 3.2 ± 15.1 at midwifery birth centers. Among hospitals, 46 were maternity units (39.0 %), 36 were maternity and gynecology unit (30.5 %) and 36 were mixed units that include maternity, gynecology and other specialties (30.5 %).

Table 2 shows the policies guiding mothers’ care during the second stage of labor. Table 3 shows ‘in what cases/how were care policies implemented’.

**Table 2:** The policies guiding mothers’ care during the second stage of labor

Variables	Hospitals *n*=118	Clinics *n*=66	Midwifery Birth Centers *n*=71	*p*
Perineal massage education during the second stage of labor				
almost all cases	10 (8.5)	5 (7.6)	15 (21.1)	.056
depending on the case	39 (33.1)	19 (28.8)	22 (31.0)	
not implemented	69 (58.5)	42 (63.6)	34 (47.9)	
not stated	0	0	0	
Active birth during the second stage of labor				
implemented	65 (56.0)	21 (31.8)	68 (100)	<.001
not implemented	51 (44.0)	45 (68.2)	0	
not stated	2	0	3	
Active birth at delivery				
implemented	36 (31.0)	11 (16.7)	61 (89.7)	<.001
not implemented	80 (69.0)	55 (83.3)	7 (10.3)	
not stated	2	0	3	
Water birth				
implemented	1 (0.9)	2 (3.0)	24 (34.3)	<.001
not implemented	115 (99.1)	64 (97.0)	46 (65.7)	
not stated	2	0	1	
Applying warm compress to the perineum				
almost all cases	2 (1.7)	0	8 (11.4)	<.001
depending on the case	30 (25.6)	13 (20.0)	35 (50.0)	
not implemented	85 (72.6)	52 (80.0)	27 (38.6)	
not stated	1	1	1	
Valsalva during pushing				
almost all cases	44 (37.9)	33 (50.0)	7 (10.0)	<.001
depending on the case	69 (59.5)	30 (45.5)	47 (67.1)	
not implemented	3 (2.6)	3 (4.5)	16 (22.9)	
not stated	2	0	1	
Hands-on technique to support fetal expulsion				
almost all cases	104 (89.7)	57 (87.7)	38 (54.3)	<.001
depending on the case	11 (9.5)	7 (10.8)	24 (34.3)	
not implemented	1 (0.9)	1 (1.5)	8 (11.4)	
not stated	2	1	1	
Perineal disinfection				
implemented	110 (94.0)	56 (84.8)	51 (72.9)	<.001
not implemented	7 (6.0)	10 (15.2)	19 (27.1)	
not stated	1	0	1	
Episiotomy for primiparas				
almost all cases	24 (20.7)	16 (24.6)	1 (1.5)	<.001
depending on the case	91 (78.4)	43 (66.2)	3 (4.4)	
not implemented	1 (0.9)	6 (9.2)	64 (94.1)	
not stated	2	1	3	
Episiotomy for multiparas				
almost all cases	2 (1.7)	0	0	<.001
depending on the case	109 (94.0)	57 (87.7)	0	
not implemented	5 (4.3)	8 (12.3)	68 (100)	
not stated	2	1	3	
Fundal pressure during the second stage of labor				
almost all cases	1 (0.9)	0	0	<.001
depending on the case	106 (91.4)	54 (83.1)	22 (32.4)	
not implemented	9 (7.8)	11 (16.9)	46 (67.6)	
not stated	2	1	3	
Hands and knees position for correcting fetal abnormal rotation				
almost all cases	10 (8.6)	5 (7.9)	16 (23.2)	<.001
depending on the case	63 (54.3)	21 (33.3)	34 (49.3)	
not implemented	43 (37.1)	37 (58.7)	19 (27.5)	
not stated	2	3	2

**Table 3:** In what cases/how were care policies implemented

Variables	*n (%)*
Perineal disinfection (*n*=217)	
washing away by antiseptic solution	152 (59.6)
spread antiseptic solution on perineum	60 (23.5)
washing away by sterilized water	10 (3.9)
washing away by tap water	4 (1.6)
not stated	1
Episiotomy for primipara (*n*=137)*	
severe distress of the fetus	129 (94.2)
prospect of severe perineal injury	120 (87.6)
vacuum/forceps delivery	111 (81.0)
others	5 (3.6)
Episiotomy for multipara (*n*=166)*	
severe distress of the fetus	149 (89.8)
prevent severe perineal injury	140 (84.3)
vacuum/forceps delivery	132 (79.5)
others	7 (4.2)
Fundal pressure (*n*=182)*	
lower fetal heart beat	117 (64.3)
poor fetus descent	108 (59.3)
vacuum/forceps delivery	99 (54.4)
stricture in area of maternal pelvic outlet	58 (31.9)
prolonged labour	52 (28.6)
others	18 (9.9)
Hands and knees position for correcting fetal abnormal rotation (*n*=149)	
depends on women’s request	98 (66.7)
15–29 min	15 (10.2)
30–59 min	28 (19.0)
more than 1 h	6 (4.1)
not stated	2

*multiple responses

#### Perineal massage education during the pregnancy

A minority of institutions routinely educated perineal massage to pregnant women: 8.5 % hospitals, 7.6 % Clinics, and 21.1 % midwifery birth centers. Around one-third of the institutions, 33.1 % hospitals, 28.8 % clinics, and 31.0 % midwifery birth centers reported ‘depending on the case’. Although more midwifery birth centers reported ‘almost all cases’ compared to hospitals and clinics, 47.9 % midwifery birth centers, 58.5 % hospitals, and 63.6 % clinics reported ‘not implemented’. There were no significant differences between each institution.

#### ’Active birth’ during the second stage

All midwifery birth centers (100 %) plus a small majority of hospitals (56.0 %) compared to a minority of clinics (31.8 %) ‘implemented’ care for women in a comfortable position. There were significant differences between each institution.

#### ‘Active birth’ at delivery

Most midwifery birth centers (89.7 %) ‘implemented’ ‘active birth’ compared to 31.0 % hospitals and 16.7 % clinics. There were significant differences between each institution.

#### Water birth

The majority 99.1 % hospitals, 97.0 % clinics, and 65.7 % midwifery birth centers reported ‘not implemented’. Although the majority of institutions reported ‘not implemented’, midwifery birth centers had significantly more water births.

#### Applying warm compress to the perineum in order to prevent perineal tears during the second stage of labor

Only a small minority, 1.7 % hospitals, 0 % Clinics, and 11.4 % midwifery birth centers reported that applying warm compress to the perineum was offered for ‘almost all cases’. Although more midwifery birth centers reported ‘almost all cases’ or ‘depending on the case’ compared to hospitals and clinics, 72.6 % hospitals, 80.0 % clinics, and 38.6 % midwifery birth centers reported ‘not implemented’.

#### Valsalva during pushing

While 37.9 % hospitals, 50.0 % clinics and 10.0 % midwifery birth centers reported that Valsalva during pushing was offered for ‘almost all cases’, a higher number 59.5 % hospitals, 45.5 % clinics, and 67.1 % midwifery birth centers reported ‘depending on the case’. Although less midwifery birth centers reported ‘almost all cases’ compared to hospitals and clinics, most midwifery birth centers reported ‘depending on the case’. Few hospitals and clinics reported ‘not implemented’.

#### Hands-on technique to support fetal expulsion

Perineal supports to protect the perineum during the birth of a baby were offered for ‘almost all cases’: 89.7 % hospitals, 87.7 % Clinics, and 54.3 % midwifery birth centers. ‘Depending on the case’ were, 9.5 % hospitals, 10.8 % clinics, and 34.3 % midwifery birth centers. Although there were significant differences between institutions, few institutions reported ‘not implemented’.

#### Perineal disinfection

A larger majority of institutions reported perineal disinfection: 94.0 % hospitals, 84.8 % clinics, and 72.9 % midwifery birth centers. Reporting ‘not implemented’ were, 6.0 % hospitals, 15.2 % clinics, and 27.1 % midwifery birth centers. Most hospitals offered perineal disinfection. Types of perineal disinfection were, washing away by antiseptic solution (59.6 %), spread antiseptic solution on perineum (23.5 %), washing away by sterilized water (3.9 %) and washing away by tap water (1.6 %). Antiseptic solutions used were invert soap (52.3 %), povidone (34.0 %), chlorhexidine (5.7 %) and others (8.0 %).

#### Episiotomies for primiparas

Only a minority of institutions routinely provided episiotomies for primiparas: 20.7 % hospitals, 24.6 % Clinics, and 1.5 % midwifery birth centers. The majority of hospitals (78.4 %) and clinics (66.2 %), compared to only 4.4 % of midwifery birth centers reported ‘depending on the case’. There were considerable differences among hospitals, clinics and midwifery birth centers. Reasons for the episiotomy were: ‘severe distress of the fetus’ (94.2 %), ‘prospect of severe perineal injury’ (87.6 %), ‘vacuum/forceps delivery’ (81.0 %), ‘others’ (3.6 %).

#### Episiotomies for multiparas

Even fewer institutions provided routine (in almost all cases) episiotomies for multiparas: 1.7 % hospitals, 0 % Clinics, and 0 % midwifery birth centers. The vast majority 94.0 % hospitals and 87.7 % clinics, reported ‘depending on the case’ compared to no midwifery birth centers. There were considerable differences between hospitals and clinics compared to midwifery birth centers with regards to multiparas. In such cases these situations were the main consideration: severe distress of the fetus (89.8 %), prevent severe perineal injury (84.3 %), vacuum/forceps delivery (79.5 %), and others (4.2 %).

#### Fundal pressure during the second stage of labor

Only a fraction of institutions provided fundal pressure during the second stage for almost all cases: 0.9 % hospitals, 0 % clinics, and 0 % midwifery birth centers. Yet 91.4 % hospitals and 83.1 % clinics did ‘depending on the cause’ compared to 32.4 % midwifery birth centers. In most cases, midwifery birth centers (67.6 %) reported ‘not implemented’ compared to a few cases in hospitals (7.8 %) and clinics (16.9 %). Fundal pressure during the second stage was used in cases of lower fetal heart beat (64.3 %), poor fetal descent (59.3 %), vacuum/forceps delivery (54.4 %), stricture in area of maternal pelvic outlet (31.9 %), prolonged labor (28.6 %) and other (9.9 %).

#### Use of hands and knees position for correcting fetal abnormal rotation during the second stage of labor

Using the hands and knees position in most cases were midwifery birth centers (23.2 %), and fewer in hospitals (8.6 %) and clinics (7.9 %). Responding ‘depending on the case’ were 54.3 % of hospitals, 33.3 % of clinics, and 49.3 % of midwifery birth centers. Although more than half of hospitals and midwifery birth centers used hands and knees position for ‘almost all/ depending on the cases’, more than half of clinics (58.7 %) did not. The duration women took a hands and knees position for correcting fetal abnormal rotation during the second stage of labor was as follows: 66.7 % depended on the woman’s request, 10.2 % for 15–29 min, 19.0 % for 30–59 min, 4.1 % for more than 1 h.

## Discussion

This study reported the most recent policies during the second stage of labor among hospitals, clinics, and midwifery birth centers in the metropolitan areas of Tokyo, Kanagawa, Saitama, and Chiba in Japan, and compared that care with the Guidelines for Midwives [13]. This research not only informs the health care community about the current state of care during second stage of labor, it is also the first research comparing three types of institutions. This research will be useful to enhance the quality of care for low-risk birth in Japan, and for future evaluation of institutions’ adherence to the Guidelines for Midwifery [13]. Considerable differences were reported in these policies among the institutions, although there were some similarities between hospitals and clinics. Important questions to consider are what factors influenced the gap between present care and recommended routine care found in the Guidelines for Midwives [13] and what are the desired future trends in Japanese antenatal care.

### Perineal massage education

Perineal massage during pregnancy is one of the effective self-care strategies used by pregnant women for preventing perineal injury at birth [21], so perineal massage must be acceptable to institutions. However, only nearly half of institutions responded ‘almost all cases’ or ‘depending on the cases’. According to a Japanese survey, antenatal face-to-face education by midwife was the most influential factor for women to implement perineal self-massage [22]. Dissemination of the Guidelines for Midwives will be a solution strategy to promote perineal massage during pregnancy.

### Women’s preferred position during labor and birth

A hallmark of midwifery practice in Japan is the philosophy and practice of ‘active birth’ and ‘expectant management’ [23], thus it is no surprise that all midwifery birth centers adopted ‘active birth’ during the second stage of labor. The traditional birthing supine position is still in evidence with a small majority of the hospitals and two-thirds of the clinics. Although there are benefits and risks of each position, there is no specific evidence that the supine position is more beneficial for women [13]; supported by the NICE guidelines [4], two systematic reviews [24, 25] and two random controlled trials [26, 27]. Another study found significant differences between women who were able to move around and those who were supine for 50 % of the time or more. Women who could assume alternative positions had shorter labors, less pain, lower requests for analgesics, less need for episiotomies and fewer problems with fetal occiput rotation [28] For that reason, supported by the evidence from Guidelines for Midwives [13], institutions should consider offering laboring women a choice of positions unless an abnormal labor dictates otherwise.

### Water birth

NICE Guidelines shows that women should be informed that there is insufficient high-quality evidence to either support or discourage giving birth in water [4], however none of the other guidelines included evidence about water births. In these results, 34 % of midwifery birth centers compared to only one hospital and two clinics implemented water births. Although NICE Guidelines recommended water birth [4], the institutions with water birth capacity in Japan are limited. Hospitals and clinics with water birth resources would be very cautious in implementing due to the risk of infection and their mixed population of low and high-risk women [29]. For this reason, the rate of implementation was quite low among hospitals and clinics compared to midwifery birth centers that only manage low risk births.

### Applying warm compresses to the perineum

According to the Guidelines for Midwives [13] there was no evidence that applying warm compresses to the perineum was effective for preventing perineal trauma. However, there was evidence that the women receiving warm compress experienced less perineal pain at post-delivery day one and two compared to the control group [13]. A more recent review suggests that warm compresses were associated with a significant decrease in 3^rd^ and 4^th^ degree tears [21]. Moreover, it was shown that no harmful outcomes occurred; therefore, warm compress to the perineum could be applied if clinicians used an appropriate temperature [13]. In this research, with two-thirds of the midwifery birth centers and only a quarter of the hospitals and clinics responding ‘almost all cases or depending on the cases’, it appears to be a widespread practice of midwives and may be more related to promoting comfort. Additional research regarding the lack of using warm compresses in clinics and hospitals should be explored.

### Valsalva while pushing

The Guidelines for Midwives [13] does not provide evidence about using Valsalva maneuver while pushing during the second stage of labor. NICE Guidelines recommends that women be informed that during the second stage they should be guided by their own urge to push [4]. Yet, more than half of clinics, one-third of hospitals, and a small percentage of midwifery birth centers responded that in almost all cases they encouraged women to push even if the women did not have the urge to push. From these results it is apparent that there are still many Japanese institutions, particularly clinics and hospitals, that are using a delivery table and midwives and physicians are encouraging women to deliver in a supine position while pushing regardless of the urge. Because Japanese clinics and hospitals accept both low and high-risk women, clinicians’ attention is necessarily diverted to high-risk patients; the low risk patients are cared for efficiently but not necessarily in a way that honors an ’active birth’.

### Hands-on technique to support fetal expulsion

Guidelines for Midwives shows that there is no evidence for the effectiveness of two different methods of perineal support (hands-poised and hands-on) used to prevent perineal tears during delivery in the lateral position, however further study is needed to take other factors into consideration such as labor positions, race, and delivery environment [13]. This is based on NICE Guidelines [4] and a systematic review [21]. In Japan, midwives traditionally apply perineal support during the birth of a baby; there are some reports, but no research, about techniques of perineal support [30]. In this study, hospitals and clinics had a high rate of implementing perineal support. On the other hand, about half of midwifery birth centers answered almost all cases, and one-third answered depending on the cases, indicating a lower implementation rate. The ‘hands-poised’ support is often understood as part of ‘active birth’. An area for further research is to document the extent to which hands-poised is associated with expectant birth practices and the subsequent outcomes.

### Perineal disinfection

According to the Guidelines for Midwives [13], there is no evidence supporting the effectiveness of disinfecting the perineum prior to delivery because the infection sites for women and newborns were no different from when disinfectant like benzalkonium chloride or chlorhexidine was used compared to tap water [4]. In this research, a large majority of all institutions disinfected the perineum. Various disinfectants were used. Due to these results, it was evident that there was little diffusion of knowledge about water as an acceptable disinfectant for the perineum and this was particularly evident in hospitals and clinics. From the point of view of women’s comfort, tap water would be more than adequate. Furthermore, because expensive disinfectants are not necessary, institutions would have the benefit of cost cutting. For these reasons tap water for disinfecting the perineum should be promoted.

### Use of episiotomies for primiparas and multiparas

Guidelines for Midwives shows that there is evidence that restrictive use of episiotomies is more beneficial to women and babies when compared to those women in the routine episiotomy group [13]. This is based on NICE Guidelines [4], Guidelines for comfortable pregnancy and childbirth [5] and a systematic review [31]. These guidelines and research recommend use of an episiotomy when it is needed for an instrumental delivery or for fetal abnormality, but not for routine use [4, 5, 31]. In this study, a minority of hospitals and clinics and an even a smaller percentage of midwifery birth centers answered ‘almost all cases’ for using an episiotomy for primiparas. The majority of hospitals and clinics responded that ‘it depended on the case’. The small percentage of midwifery birth centers that responded ‘it depended on the case’ was not surprising, as midwives generally do not perform episiotomies [23]. It is possible that hospitals and clinics are performing more instrumental deliveries related to the mixed patient load of low and high-risk mothers. However, the rate of ‘almost all cases’ meant that routine use of episiotomy was higher than expected. This result might be related to conventional enforcement of episiotomies, or lack of doctors. Because it is clear that the routine use of episiotomy is unnecessary, it is important that the spread of knowledge include both midwives and doctors.

Moreover, for multiparas, most of the hospitals and clinics performed episiotomies ‘depending on the case’. From these results, the routine use for multiparas was very small compared to primiparas hence both hospitals and clinics are in accordance with the Guidelines for Midwives [13]. Furthermore, almost all institutions performed episiotomies under the appropriate circumstances such as, severe distress of fetus, to prevent severe perineal injury, and use of vacuum, or forceps. The midwifery birth clinics reported not using episiotomies for multiparas. That approach is in line with the philosophy of expectant management in midwifery.

### Fundal pressure during the second stage

Guidelines for Midwives found that there was no robust evidence available for the effects of manual fundal pressure [13]. This conclusion was based on one high quality study regarding the efficacy of using fundal pressure [32] and on the Guidelines for obstetrical practice in Japan [2]. The authors concluded that not enough high quality research has been conducted to either recommend or not recommend using fundal pressure [32]. Moreover, there is a risk of uterine rupture [33], anal sphincter damage [34] and severe perineal lacerations [35, 36]. However, the Guidelines for Obstetrical Practice in Japan recommended complementary use of fundal pressure for vacuum extraction or forceps but with caution [2]; therefore, fundal pressure should not be practiced for normal delivery. In the results of this study, only one hospital and no clinics or midwifery birth centers answered ‘used fundal pressure in almost all cases’. The vast majority of the hospitals and clinics and about one-third of the midwifery birth centers used fundal pressure depending on the case. It seems apparent that the danger of routine fundal pressure during the second stage is widely known at these institutions. However, one hospital continued to enforce routine fundal pressure during the second stage so that the current evidence has to be more widely disseminated. One would expect hospitals and clinics to use fundal pressure, depending on the case, because of instrument delivery or fetal abnormality. Specifics about the conditions under which it was used varied but all were related to complications of delivery.

### ‘Active birth’

Guidelines for Midwives recommends ’active birth’ at delivery and that a woman should be informed about both the benefits and risks of the various birthing positions; she should be able to choose the position [13]. Almost all of the midwifery birth centers practiced ’active birth’ at the delivery compared to a minority of hospitals and clinics. Given that hospitals and clinics are also caring for high-risk women with complications it is assumed that staff do not have the time to provide the extra explanation for alternative birthing positions. Other factors may also be influencing the decisions such as convention, lack of experience with the positions and lack of cooperation. Future research should investigate the reasons why few hospitals and clinics implement women’s chosen position at delivery.

### Hands and knees position to correct fetal abnormal rotation

In this study a minority of institutions implemented hands and knee position in all cases but with midwifery leading by twice as much. The tendency was to offer hands and knee position depending on the case or not at all; for example more than half of the clinics did not offer it at all. Guidelines for Midwives [13] shows that there is no obvious evidence that the hands and knees position resolves the abnormal rotation of the fetus or that it is effective in relieving the back pain that comes from abnormal rotation [13]. However, there are not so many studies of this issue so more research is needed in the future. This was the conclusion of the Guidelines for Midwives [13] based on the NICE Guidelines [4] and a systematic review [37]. NICE Guidelines, which used only one study, indicated that there was no significant difference in the number of fetuses presenting as occipitoposterior to transition to an occipitoanterior presentation [4]. A more recent study concluded that alternate positions positively and significantly influenced fetal occiput presentation [28]. Therefore, although it is necessary to conduct more research about the effects of taking hands and knees position for correcting fetal abnormal rotation during the second stage of labor it could be safely encouraged for low-risk women.

### The gap between the care policies of institutions and the Guidelines

We found some gaps between the care policies of institutions and the Guidelines. Therefore, we explored the possible reasons. Three reasons were noted: (1) differences in staff scope of practice and needs among institutions; (2) persistence of Japanese traditional practice; (3) evidence which was not supported and more research needed.

1. There were differences about staff scope of practice and needs among institutions. For example, doctors, midwives, and nurses work within a hospital, however their scope of practice is very different (e.g. episiotomy and suturing are restricted to physicians). Midwifery birth centers supported expectant or physiological management of labor and active birth. Also, because Japanese hospitals need to care for both high-risk and low-risk births, the responsibility and complexity of care within hospitals is greater [38], and hospitals tend to conduct excessive medical interventions (e.g. valsalva, episiotomy, fundal pressure). To reduce this first gap, institutions, especially hospitals need to work out systematic countermeasures so that staff resources are used to greatest advantage.
2. The gap made by Japanese traditional treatment (perineal disinfection, and hands-on technique to support fetal expulsion), or non-traditional treatment (applying warm compress to the perineum). Although the majority of hospitals, clinics and midwifery birth centers attempted to cleanse the perineum, there was a wide variation in methods. Needed is systematic diffusion of information to physicians, nurses and infection prevention committees about using only water for perineal disinfection. Applying warm compresses to the perineum is easy and not harmful to women and newborns therefore it can confidently be adopted as usual care. It is likely that if more clinicians knew of the relaxing effects of warm compresses to the perineum this care would be adopted.
3. There was no strong evidence to support water birth, and taking hands and knees position for correcting fetal abnormal rotation and more research is needed. Accumulating additional research in the Japanese context may be necessary to provide the evidence that will support building a consensus.

Study Limitations. This research has a number of limitations; first, the survey targeted only occupations or positions that knew about the care policy among hospitals, clinics, and midwifery birth centers. Therefore we cannot assume that the results of this survey indicate that all policies were translated into practice. Surveys that compare actual practice are needed. While the high response rate of the midwifery birth centers provided higher generalizability for that group, the low response rate particularly of the clinics was a limitation. Moreover, the respondents were not required to add their profession such as physicians or midwives; therefore we were unable to determine whether or not profession itself influenced the response. There is some possibility that these data reflected policies of many independent midwives. The survey tool should be subjected to additional psychometric development beyond face validity and updated, as new Cochrane reviews are available. The Midwifery Guidelines [13] will be revised, as newer research is available (e. g. NICE updated in 2014 [39] to replace the 2007 NICE [14], and the Clinical Guidelines for Obstetrical Practice in Japan were updated in 2014 [40]). In addition, a national survey is needed to understand antenatal care for the second stage of labor in the various regions of Japan.

## Conclusions

This survey has provided current information about Japanese policies during the second stage of labor. There existed considerable differences among hospitals, clinics, and midwifery birth centers practice. There were also gaps between the Guidelines for Midwifery [13] and care policies, and reasons for these gaps were discussed. Therefore new strategies to align the care policies with the Guidelines for Midwifery [13] are needed. That data will be useful for developing a plan for improve the antenatal care in Japan. Furthermore, this survey will be useful for evaluating the adherence to the Guidelines for Midwives [13] for the second stage labor in the future.

## Abbreviations

WHO: World Health Organization (WHO)
NICE: National Institute for Health and Care Excellence
Guidelines for Midwives: 2012 evidence-based Guidelines for midwifery care during childbirth
